# Effects of Extracts and Flavonoids from *Drosera rotundifolia* L. on Ciliary Beat Frequency and Murine Airway Smooth Muscle

**DOI:** 10.3390/molecules27196622

**Published:** 2022-10-05

**Authors:** Alexander Hake, Frank Begrow, Verena Spiegler, Nico Symma, Andreas Hensel, Martina Düfer

**Affiliations:** 1 Institute of Pharmaceutical and Medicinal Chemistry—Pharmacology, University of Münster, 48149 Münster, Germany; 2 Institute of Pharmaceutical Biology and Phytochemistry, University of Münster, 48149 Münster, Germany

**Keywords:** *Drosera rotundifolia*, antispasmodic, trachea, ciliary beat frequency, phosphodiesterase

## Abstract

Extracts from *Drosera rotundifolia* are traditionally used to treat cough symptoms during a common cold. The present study aimed to investigate the impact of extracts from *D. rotundifolia* and active compounds on the respiratory tract. Tracheal slices of C57BL/6N mice were used ex vivo to examine effects on airway smooth muscle (ASM) and ciliary beat frequency (CBF). Phosphodiesterase (PDE) inhibition assays were carried out to test whether PDE1 or PDE4 are targeted by the active compounds. An ethanol–water extract, as well as an aqueous fraction of this extract, exerted antispasmodic properties against acetylcholine-induced contractions. In addition, contractions induced by 60 mM K^+^ were abrogated by the aqueous fraction. Effects on ASM could be attributed to the flavonoids quercetin, 2″-*O*-galloylhyperoside and hyperoside. Moreover, the *Drosera* extract and the aqueous fraction increased the CBF of murine tracheal slices. Quercetin and 2″-*O*-galloylhyperoside were identified as active compounds involved in the elevation of CBF. Both compounds inhibited PDE1A and PDE4D. The elevation of CBF was mimicked by the subtype-selective PDE inhibitor rolipram (PDE4) and by 8-methoxymethyl-IBMX. In summary, our study shows, for the first time, that a *Drosera* extract and its flavonoid compounds increase the CBF of murine airways while antispasmodic effects were transferred to ASM.

## 1. Introduction

*Drosera rotundifolia* L. is a carnivorous plant that natively grows in peatland or other wet and oligotrophic regions of the world [1]. The medicinal use to treat symptoms of a common cold, especially to treat a dry cough, is due to tradition and belongs to the area of complementary medicine. The extraction process of the fresh undried herbal drug is defined by the European Pharmacopoeia leading to the mother tincture, which serves as the raw material for medicinal drugs available in Germany [2,3]. In addition to *D. rotundifolia*, *D. anglica* and *D. intermedia* are of pharmaceutical relevance [3].

The phytochemical composition of *D. rotundifolia* is well characterized and consists of a complex mix of flavonoids, mainly flavonols and their glycosides (e.g., quercetin, isoquercitrin, hyperoside and 2″-*O*-galloylhyperoside) [4,5]. 2″-*O*-galloylhyperoside is one flavonoid of high relevance because of the infrequent occurrence in the plant kingdom compared to quercetin or isoquercitrin. 1,4-Naphtoquinones described for *D. rotundifolia* are particularly the main naphthoquinone 7-methyljuglone as well as plumbagin, hydroxydroserone, ramentone and their glycosides [6,7]. Other compounds reported are ellagic acid and dimethyl ellagic acid, as well as anthocyanins [4,6].

From the pharmacological point of view, studies focused on ex vivo experiments investigating antispasmodic effects and anti-inflammatory and antibacterial properties [8,9,10,11,12,13]. In addition to functional aspects, recent studies also provide evidence for the influence of *Drosera* on gene expression [14]. Despite the fact that the indication area of extracts from *Drosera* spp. addresses the respiratory tract, many ex vivo studies were performed using intestine smooth muscle [8,9,10]. Until now, effects on the lower respiratory tract have been insufficiently studied. Of note, a phytochemically uncharacterized ethanolic extract of *Drosera* was reported to have only a slightly antispasmodic effect on guinea pig tracheal smooth muscle, as not more than 15% of a carbachol-induced contraction of the trachea could be abrogated [10]. The same extract exerted much stronger effects on intestine muscles, inhibiting almost 50% of acetylcholine-induced contractions [10]. In the same line, an ethanolic extract of *D. madagascariensis* was ineffective against PGF_2_-induced contractions on guinea-pig tracheal slices [8].

Considerably better understood are the anti-inflammatory properties of *Drosera*, as extracts of *D. rotundifolia* and *D. madagascariensis* and their natural compounds (quercetin, isoquercitrin and hyperoside) turned out to be inhibitors of the neutrophil elastase [8,9]. In addition, extracts of *D. rotundifolia* and *D. madagascariensis* and their flavonoids were potent anti-inflammatory agents in a HET-CAM assay [11], and fractions of *D. rotundifolia* and *D. tokaiensis* were reported to suppress the activation of human mast cells [12]. Antibacterial activity against oral bacteria was reported for extracts of *D. peltata,* pointing out that plumbagin is the active component [13].

To the best of our knowledge, no experiments have been performed examining the influence of extracts from *Drosera* on ciliary beat frequency (CBF), ciliary beating amplitude (CBA) or mucociliary clearance until now. The mucociliary clearance is a major host defense mechanism of the upper and lower airways, which is responsible for eliminating inhaled particles of dust or pathogens [15,16,17] and thereby represents a powerful target to assess. Parameters with an impact on mucociliary clearance are particularly the CBF and CBA [15,16,18]. Certain spasmolytics used for the treatment of chronic obstructive pulmonary disease (COPD) and bronchial asthma were reported to increase the CBF of airway epithelium cells. For instance, salbutamol, a β_2_ adrenergic agonist, increases ciliary beating by rising cAMP levels [16,19]. Furthermore, the phosphodiesterase 4 (PDE 4) inhibitor roflumilast-N-oxide, which is the active metabolite of roflumilast, was reported to increase CBF of cultured bronchial epithelium cells via a rise in cAMP [17,20].

The present study provides a detailed investigation of the effects of extracts and natural compounds from *D. rotundifolia* on airway smooth muscle (ASM) and CBF. Characterization of the ethanolic extract was performed by HPLC-MS. Murine tracheal slices were used to examine the influence on smooth muscle contractility and ciliary beating of ciliated tracheal epithelium cells. Further experiments were carried out with single compounds to test for an influence on PDE1A and PDE4D.

## 2. Results

### 2.1. Phytochemical Characterization of Extracts and Quantification of Flavonoids

**Figure 1:**
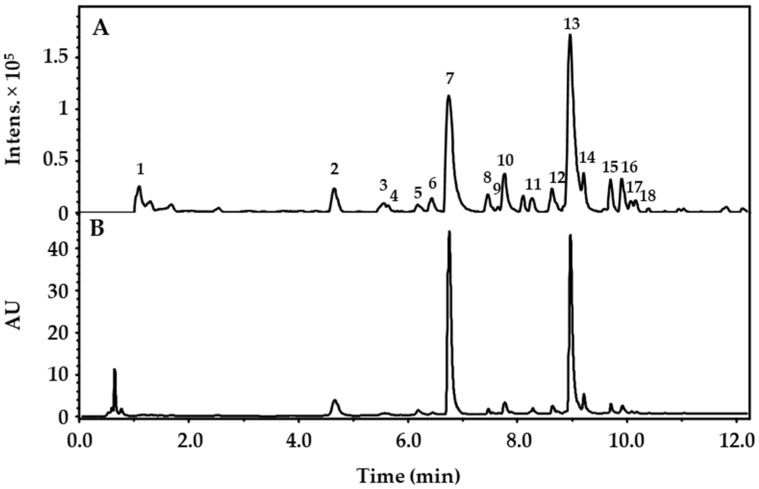
Base peak chromatogram (**A**) and UV chromatogram at *λ* = 280 nm (**B**) of the *Drosera* dry extract (DE). Identified compounds are represented by numbers as classified in Table 1.

The fresh undried herbal material of *Drosera rotundifolia* was extracted with ethanol:water (9:1, *v/v*), as described in detail in the methods for the preparation of homeopathic stocks and potentisation (method 1.1.3) of the European Pharmacopoeia [2]. After the removal of ethanol in vacuo, the remaining aqueous extract was lyophilized, yielding the *Drosera* dry extract. For the experiments, the lyophilisate was either fully solved (“*Drosera* extract”, DE) or an aqueous fraction (“*Drosera* fraction, aqueous”, DF_A_) was generated by suspending the lyophilisate in Krebs–Henseleit buffer and removing insoluble components (see methods, 4.2, for details). DE was characterized by UHPLC-(+)-ESI-qTOF-MS, and peaks were related to known natural compounds described for *Drosera* spp. (Table 1 and Figure 1). The amount of the flavonoids quercetin, hyperoside and 2″-*O*-galloylhyperoside was determined by UHPLC-PDA (Table 2) for DE (acetonitrile/water, 1:1, *v/v*) and for DF_A_.

### 2.2. Effects of the Drosera Extract, the Aqueous Drosera Fraction and Isolated Flavonoids on ASM Contractions

**Figure 2:**
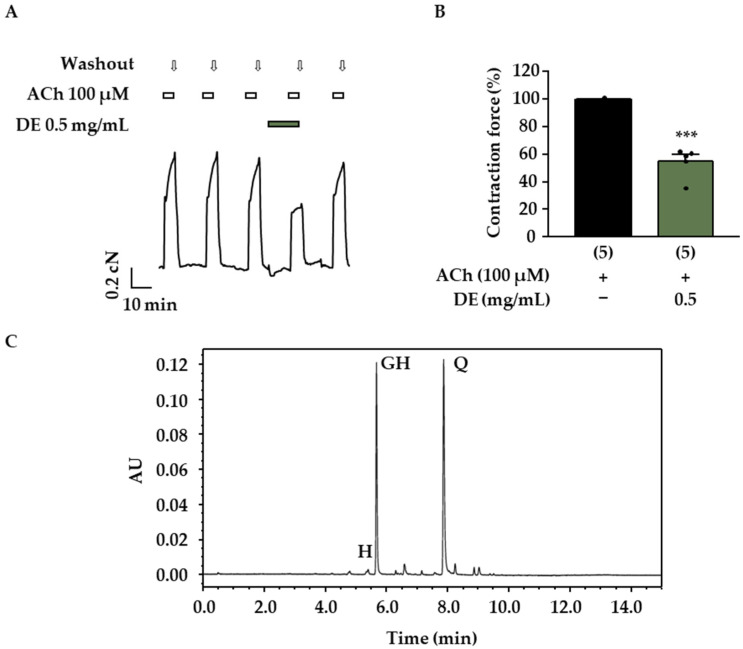
Influence of DE on the ACh-induced contraction of mouse tracheal slices. (**A**) Representative measurement examining the antispasmodic activity of DE on the pharmacomechanical coupling. At least 3 ACh-induced control contractions (100 µM ACh) were recorded before DE (0.5 mg/mL) was added to the bath solution for a 10-minute pretreatment period. Thereafter, another contraction was induced by ACh. In the presence of DE, the contraction force was significantly reduced. Reversibility was proven by a washout period followed by another ACh bolus. (**B**) The scatterplot illustrates the mean contraction force in % ± SD (related to the control contraction) and single data points of 5 independent mouse preparations. (**C**) UV chromatogram of DE at *λ* = 360 nm. 2″-*O*-galloylhyperoside (GH), quercetin (Q) and hyperoside (H) are labeled as the characteristic flavonoids contained in the extract. (***: *p* < 0.001 vs. control).

**Figure 3:**
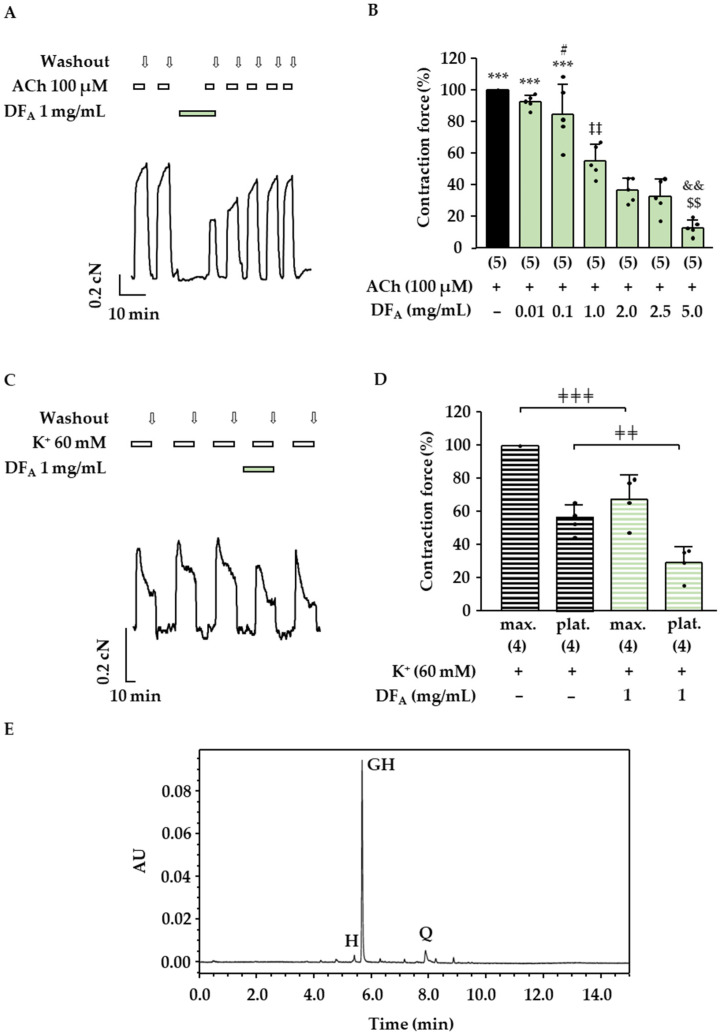
Influence of DF_A_ on the ACh-induced contraction of mouse tracheal slices. (**A**) Representative measurement investigating the effect on the ACh-induced contraction of ASM. First, ACh-induced control contractions (100 µM ACh) were induced. Afterward, DF_A_ (1 mg/mL) was added to the bath solution for a pretreatment time of 10 min, and another contraction was induced by ACh in the presence of DF_A_. (**B**) The scatterplot represents the decrease in the mean contraction force in % ± SD in response to treatment with DF_A_ (5 independent mouse preparations). (**C**) Representative measurement to investigate the influence on the 60 mM K^+^-induced contractions of ASM. At least 3 constant contractions evoked by K^+^ were recorded. Afterward, DF_A_ was added for 10 min, and K^+^ was applied again. This maneuver was followed by a washout phase to check for reversibility. (**D**) The scatterplots illustrate the mean contraction maximum (max.) and the plateau (plat.) in % ± SD (related to the respective control condition) and the single data points of four independent mouse preparations. (**E**) UV chromatogram at *λ* = 360 nm of DF_A_ at a concentration of 0.5 mg/mL. 2″-*O*-galloylhyperoside (GH), quercetin (Q) and hyperoside (H) are labeled as characteristic flavonoids contained in the extract. (#: *p* ≤ 0.05 vs. control contraction, ***: *p* < 0.001 vs. DF_A_ ≥ 1.0 mg/mL, ‡‡: *p* < 0.01 vs. ≥ DF_A_ 1 mg/mL, &&: *p* < 0.01 vs. DF_A_ 2 mg/mL, $$: *p* < 0.01 vs. DF_A_ 2.5 mg/mL, ╪╪╪: *p* < 0.001, ╪╪: *p* < 0.01).

In order to systematically investigate the antispasmodic activity of *D. rotundifolia* on the trachea, first, the influence of DE and DF_A_ on tracheal contractions induced by stimulation of muscarinic receptors with acetylcholine (ACh, 100 µM) was addressed. DE and DF_A_ were added 10 min before and during the application of the cholinergic stimulus in these experiments. Both preparations showed antispasmodic effects (Figure 2A,B and Figure 3A,B). The contraction maximum evoked by 100 µM ACh was reduced to 55 ± 11% (*p* < 0.001, Figure 2B) by 0.5 mg/mL DE (Krebs–Henseleit buffer, 1% DMSO).

**Figure 4:**
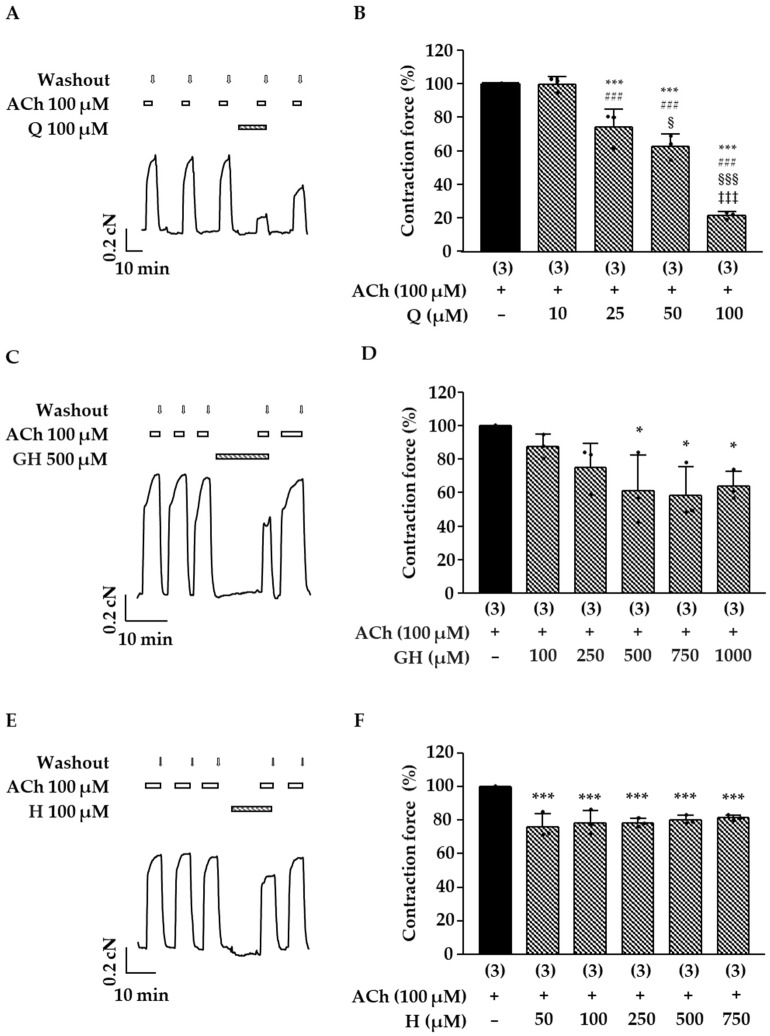
Influence of flavonoids contained in *D. rotundifolia* on ACh-induced contractions of mouse tracheal slices. Experiments were performed as described in Figure 2A. (**A**,**C**,**E**) Representative recordings are shown. Quercetin (Q, 100 µM), 2″-*O*-galloylhyperoside (GH, 500 µM) and hyperoside (H, 100 µM) were added to the bath solution for a period of 10 min preceding the next bolus of ACh (100 µM). (**B**,**D**,**F**) The scatterplots represent the single data points and the mean contraction force in % ± SD of 3 independent mouse preparations. (*: *p* ≤ 0.05 and ***: *p* < 0.001 vs. control contraction, ###: *p* < 0.001 vs. Q 10 µM, §: *p* < 0.05 and §§§: *p* < 0.001 vs. Q 25 µM, ‡‡‡: *p* < 0.001 vs. Q 50 µM).

Similar results were obtained with the double dose of the more hydrophilic fraction DF_A_ (Figure 3A,B: contraction force DF_A_ 1.0 mg/mL + ACh 55 ± 9%, related to the control with ACh, which was set to 100%; *p* < 0.001). Further experiments revealed a clear concentration–response correlation for DF_A_ and an IC_50_ value of 1.1 mg/mL (CI: 0.8–1.4 mg/mL) was calculated. These data show that the compounds solubilized in DE and DF_A_ affect the pharmacomechanical coupling of mouse trachea. Next, the influence of DF_A_ on the electromechanical coupling was investigated. After a pretreatment period of 10 min, the contraction force induced by 60 mM K^+^ was significantly decreased in the presence of 1 mg/mL DF_A_ (Figure 3C,D). In this series of experiments, the initial peak and the plateau phase of the K^+^-mediated contractions were evaluated, revealing that both parameters were affected by DF_A_. The initial K^+^-induced peak was reduced to 68 ± 15% of the control contraction maximum without DF_A_ (*p* < 0.01). The contraction plateau amounted to 30 ± 10% after treatment with DF_A_ (*p* ≤ 0.05 vs. control contraction plateau). The chromatograms presented in Figure 2C and Figure 3E show that 2″-*O*-galloylhyperoside is a major flavonoid in both preparations, whereas quercetin is an additional main component of DE. In order to assess the relevance of these compounds for the effect of DE and DF_A_, they were tested on ACh-induced contractions of the mouse trachea. Hyperoside, a flavonoid contained in the dry extract of *D. rotundifolia* (Table 1) in low amounts, is structurally closely related to quercetin and 2″-*O*-galloylhyperoside and was investigated in the same manner. Quercetin exerted a dose-dependent antispasmodic effect (Figure 4A,B) and decreased the effect of 100 µM ACh to 74 ± 11% (25 µM quercetin, *p* < 0.001 vs. ACh alone), 63 ± 7% (50 µM quercetin, *p* < 0.001) and 21 ± 2% (100 µM quercetin, *p* < 0.001). In the case of 2″-*O*-galloylhyperoside and hyperoside, no dose-dependency was observed. The ACh-induced contractions were significantly inhibited by 500 µM 2″-*O*-galloylhyperoside and reduced to 61 ± 21% of control (*p* ≤ 0.05 vs. ACh alone), while higher concentrations (750 and 1000 µM) did not significantly differ from the aforementioned effect (Figure 4C,D). Hyperoside (50 to 750 µM) had the smallest effect of the three flavonoids and decreased ACh-mediated contraction force to approximately 80% of control at all concentrations tested (Figure 4E,F).

### 2.3. Influence of DE and Flavonoids on CBF

**Figure 5:**
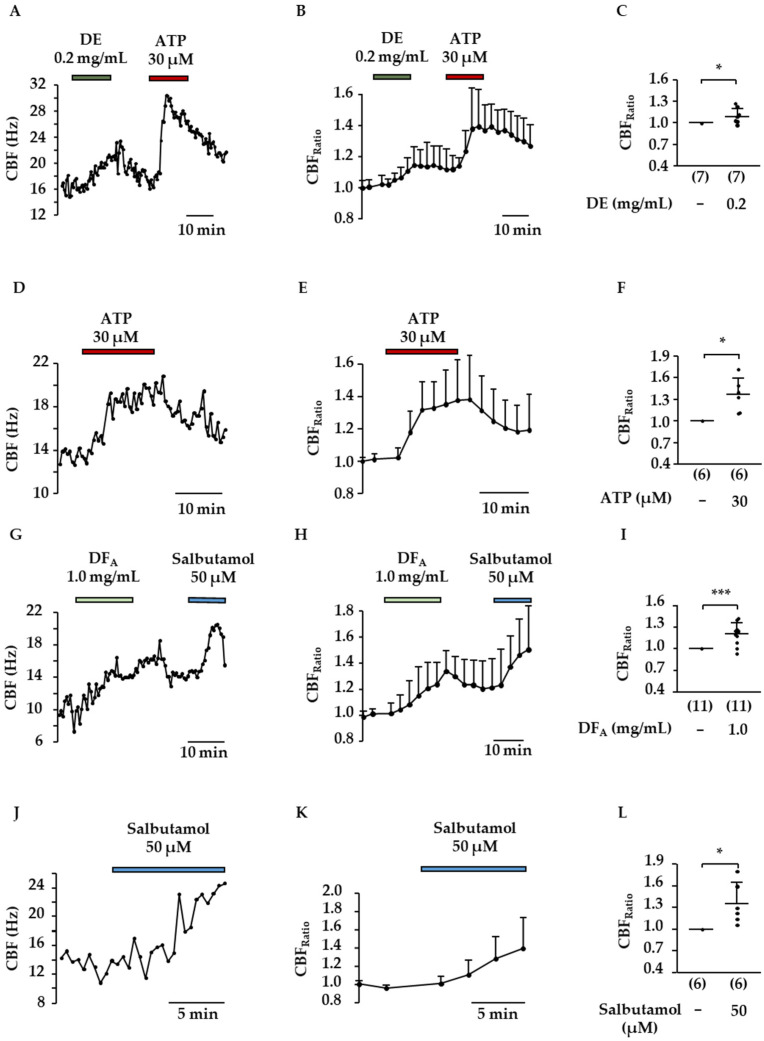
Influence of DE and DF_A_ on the CBF of ciliated tracheal epithelium cells. In (**A**,**D**,**G**,**J**), representative measurements are shown. After determination of the basal CBF test solutions containing DE (0.2 mg/mL), DF_A_ (1.0 mg/mL), ATP (30 µM) or salbutamol (50 µM) were added for 15, or in case of salbutamol for 10 min. The sequence of treatments and washout periods was performed as indicated in the exemplary recordings. Graphs (**B**,**E**,**H**,**K**) represent the averaged time-dependent change in CBF normalized to the basal CBF for each experiment. The scatterplots (**C**,**F**,**I**,**L**) illustrate the mean CBF_Ratio_ ± SD, related to CBF_Basal_. Here the CBF_Ratio_ represents the averaged values in minutes 10 to 15 and, in the case of salbutamol, in minutes 5 to 10 after changing to the aforementioned test solutions. The number of experiments is shown in the brackets. (***: *p* < 0.001, *: *p* ≤ 0.05).

The influence of DE and DF_A_ on CBF was measured using murine tracheal slices cultured for 1 to 7 days. Test solutions and compounds were added during constant perfusion (1 mL/min). ATP (30 µM) or salbutamol (50 µM) were applied at the end of each experiment as positive controls and both substances increased the CBF as expected (Figure 5F: CBF_Ratio-ATP_ = 1.4 ± 0.2, *p* ≤ 0.05 compared to CBF_Basal_; Figure 5L: CBF_Ratio-Salbutamol_ = 1.4 ± 0.3, *p* ≤ 0.05 compared to CBF_Basal_). Both *Drosera* preparations significantly increased the CBF of ciliated tracheal epithelial cells (Figure 5A–C,G–I). The more lipophilic DE elevated the CBF by approximately 10% already at a concentration of 0.2 mg/mL (Krebs–Henseleit buffer, 0.3% DMSO) (Figure 5C: CBF_Ratio_ = 1.1 ± 0.1, *p* ≤ 0.05 vs. CBF_Basal_). DF_A_ was tested at a concentration of 1 mg/mL and induced a rise in CBF of 20% on average (Figure 5I: CBF_Ratio_ = 1.2 ± 0.2, *p* < 0.001 vs. CBF_Basal_).

**Figure 6:**
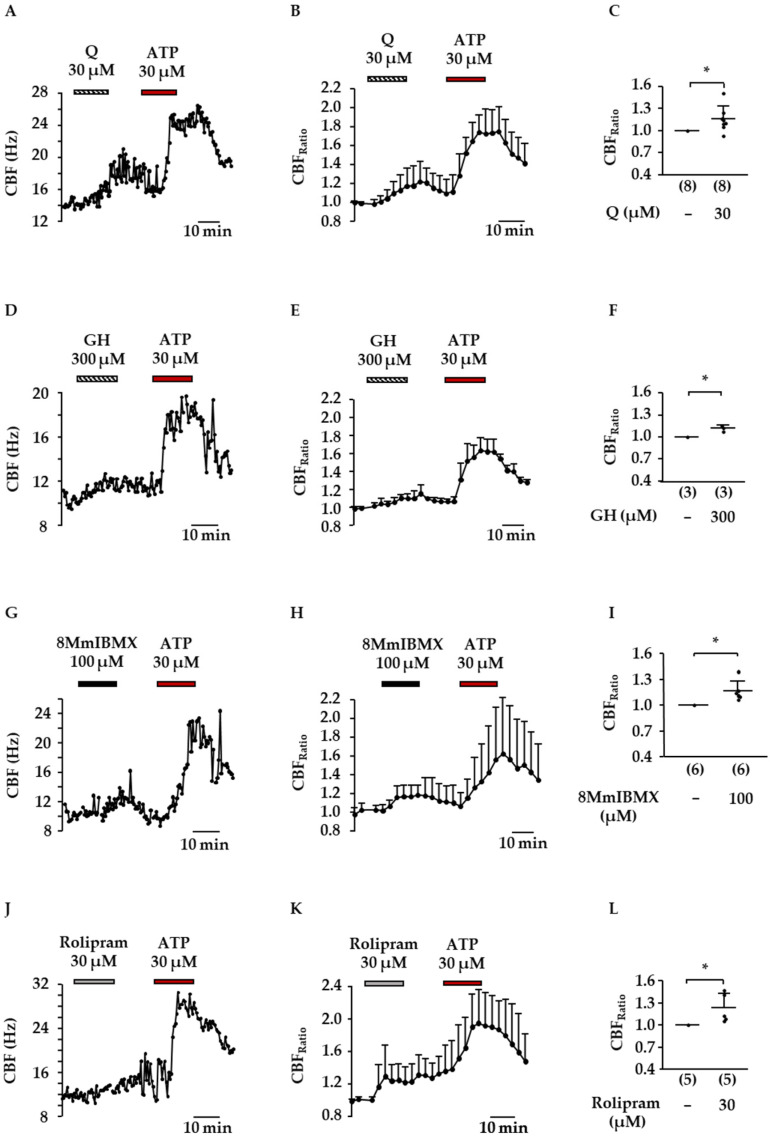
Influence of flavonoids from *D. rotundifolia* on the CBF of ciliated tracheal epithelial cells. In (**A**,**D**,**G**,**J**), representative example measurements are illustrated. In analogy to the other measurements, the basal CBF was recorded before changing to test solutions. Quercetin (Q), 2″-*O*-galloylhyperoside (GH), 8MmIBMX and rolipram were added for 15 min. Afterward, test substances were removed, and the positive control ATP (30 µM) was added, followed by another washout period. In (**B**,**E**,**H**,**K),** the time-dependent change in CBF normalized to CBF_Basal_ is illustrated in 2.5 min steps. The scatterplots (**C**,**F**,**I**,**L**) illustrate the mean CBF_Ratio_ ± SD related to CBF_Basal_. The CBF_Ratio_ represents the averaged values in minutes 10 to 15 after changing to the aforementioned test solutions. The number of experiments is represented in the brackets. (*: *p* ≤ 0.05).

Effects in the same order of magnitude were observed by the flavonoids quercetin (30 µM, Figure 6A–C) and 2″-*O*-galloylhyperoside (300 µM, Figure 6D–F). In the presence of quercetin, the CBF_Ratio_ amounted to 1.2 ± 0.2 (*p* ≤ 0.05 vs. control), and a tenfold higher concentration of 2″-*O*-galloylhyperoside increased the CBF_Ratio_ to 1.11 ± 0.04 (*p* ≤ 0.05 vs. control). It is known that cAMP and cGMP play an important role in the regulation of CBF. Due to the fact that quercetin was reported to inhibit PDE 1–5, two PDE inhibitors were used to elucidate whether the subtypes PDE1A and PDE4 could mimic the effects observed for flavonoids and preparations of *Drosera* dry extract. Both the PDE1A selective inhibitor 8-methoxymethyl-(8Mm)IBMX (Figure 6G–I) and the PDE4 selective inhibitor rolipram (Figure 6J–L) significantly increased the CBF by 20% on average (CBF_Ratio_ (8MmIBMX, 100 µM): 1.2 ± 0.1, CBF_Ratio_ (rolipram_,_ 30 µM): 1.2 ± 0.2, *p* ≤ 0.05 compared to CBF_Basal_ for both inhibitors).

### 2.4. Inhibition of PDE1A and PDE4D by Flavonoids Present in D. rotundifolia

**Figure 7:**
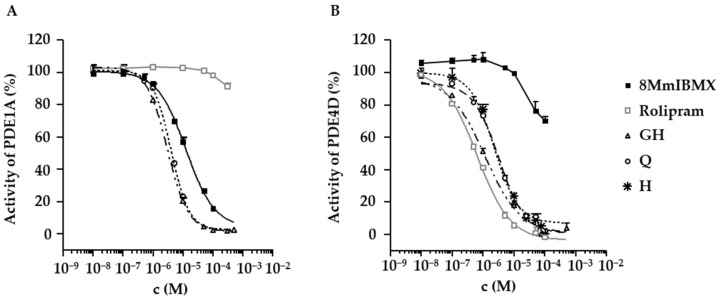
Influence of flavonoids from *D. rotundifolia*, 8MmIBMX and rolipram on the activity of the PDE1A (**A**) and PDE4D (**B**). 2″-*O*-galloylhyperoside (GH), quercetin (Q), hyperoside (H) and the respective positive controls 8MmIBMX and rolipram were tested on the activity of the PDEs in a cell-free assay. Samples were measured as duplicates. Enzyme activity is given in % ± SD and refers to the control without inhibitor.

In order to verify the proposed targets of the natural compounds contained in the DE, two PDE inhibitor assays were performed. As expected, quercetin reduced the in vitro activity of the PDE1A and PDE4D (Table 3 and Figure 7). The 2″-*O*-galloylhyperoside and hyperoside, which to our knowledge have not been described as inhibitors of the PDE until now, turned out to inhibit the PDE4D with IC_50_ values in the low µM range (Table 3 and Figure 7B). The 2″-*O*-galloylhyperoside was also tested for its inhibitory potency on the PDE1A (Table 3 and Figure 7A) and was shown to be very effective on this PDE as well. Rolipram did not have an influence on the activity of PDE1A up to a concentration of 100 µM (PDE1A activity: 98.2 ± 0.6%) and only slightly inhibited PDE1A at 300 µM (PDE1A activity: 92 ± 2%). Regarding PDE4D, rolipram inhibited this PDE in a dose-dependent manner as expected with an IC_50_ below 1 µM. Interestingly, 8MmIBMX lost its selectivity for PDE1A and influenced PDE4D already at a concentration of 50 µM (PDE4D activity with 50 µM 8MmIBMX: 77 ± 6%, with 100 µM 8MmIBMX: 70 ± 2%).

## 3. Discussion

*Drosera* extracts are traditionally used to treat common cold symptoms, especially due to proposed antispasmodic activities. The inhibition of PDEs, especially the inhibition of PDE3 and PDE4, are known targets for airway dilatation by rising cAMP and cGMP levels [22,23,24]. Thus, it is likely, that the effects observed are due to an inhibition of the PDEs by the flavonoids. Data supporting the latter are given by the PDE inhibitor assays. It has to be taken into account that there is a different response to sub-selective PDE inhibitors in different organ tissues [23,25], exemplifying the limits of using intestine smooth muscle as a model for drugs addressing the lower respiratory tract. In previous experiments, *Drosera* extracts were reported to exhibit rather weak relaxing effects on ASM using guinea-pig tracheal slices [8,10], while current results imply strong effects, as ACh- and K^+^-induced contractions were significantly abrogated.

Interestingly, in the present study, quercetin failed to abrogate ACh-induced contraction on ASM at a concentration of 10 µM (Figure 4), while this concentration was reported to inhibit carbachol-induced contractions on guinea-pig ileum by more than 50% underlining the aforementioned tissue-specific differences [26]. These divergences in antispasmodic potency on different organ tissues might explain weak effects on guinea-pig ASM and the strong effects on guinea-pig ileum reported for *Drosera* extracts in the past. The tissue specificity of plant extracts and natural compounds are indeed of high relevance. For instance, a tissue specific antispasmodic activity on uterine and intestine smooth muscle has been reported for an extract of *Marrubium vulgare*, whereas it was absent in ASM [27]. Regarding the active compounds, current results coincide with the literature, as in earlier studies on intestine smooth muscle, antispasmodic effects were related to the flavonoids quercetin and isoquercitrin [9,26]. In line with these previous findings, the dry extract used within this study was pharmacologically active despite the absence of naphthoquinones [9], which have formerly often been regarded as the active principle in *Drosera* extracts and considered as marker compounds for quality control [28]. These findings are certainly favorable with respect to the general toxicity of this compound class [29,30].

Our study showed that quercetin and its glycosides were responsible for the antispasmodic activities on ASM, with quercetin being the most potent substance (Figure 4). Effects of quercetin on murine tracheal slices and the potent inhibition of PDE4D concur with a previous study, which demonstrated that its antispasmodic property is mediated via dual inhibition of PLCβ and PDE4 [31]. For 2″-*O*-galloylhyperoside, a flavonoid that is described to exert anti-inflammatory properties [32], it is the first time that an antispasmodic activity on ASM could be demonstrated. Hyperoside, which only appears in very low amounts, turned out to be less potent on the maximal effect against ACh-induced contractions on ASM and reduced the contraction force by maximally 20–25% (Figure 4). In the literature, only antispasmodic effects on KCl-induced (80 mM) contractions have been reported thus far [33]. Nevertheless, in synergy with the other flavonoids, it might contribute to the antispasmodic effects under cholinergic stimulation. As shown in earlier studies, the glycosides were less potent than the respective aglycon, which might be explained by their hydrophilicity resulting in a decreased ability to penetrate cell membranes [34,35]. Differences in PDE selectivity, as were reported for luteolin and luteolin-7-*O*-glycoside [34], are also possible.

In addition to the antispasmodic properties of *Drosera* extracts and flavonoids, their effects on mucociliary clearance are of high relevance. Previous studies demonstrated that increasing the CBF has a crucial influence on mucociliary clearance; for instance, an increase in CBF by 16% results in an increase in mucociliary clearance by even 56% [18]. Our data show that the CBF of ciliated tracheal epithelium cells was significantly increased by low amounts of DE (0.2 mg/mL) and by the aqueous *Drosera* fraction (1.0 mg/mL). Quercetin and 2″-*O*-galloylhyperoside were identified as active compounds. Their effects could be mimicked by the PDE inhibitors rolipram and 8MmIBMX, which are already known to increase ciliary beating [36,37]. PDE1A was shown to be located directly in the cilia [36]. The PDE inhibition assays demonstrate that, besides quercetin, which was already characterized as an inhibitor of PDE 1–5 [34], 2″-*O*-galloylhyperoside effectively targets PDE1A and 4D. Certainly, it cannot be excluded that inhibition of other PDEs is involved as well. While an increase in the ciliary beating of both tracheal epithelium and lung epithelium cells was described for PDE4 inhibitors by various groups, the role of PDE1A was only addressed in lung epithelium cells [36,37] but not in the trachea. Our experiments with 8MmIBMX indicate that PDE1A also regulates the activity of tracheal cilia. Of note, Figure 7 illustrates that 8MmIBMX is not fully selective. In consequence, acceleration of CBF by 8MmIBMX cannot be definitely assigned to PDE1A inhibition. Nevertheless, for the two flavonoids tested, the effects on ciliated epithelium cells of the lower respiratory tract are shown for the first time. Quercetin was reported to increase cystic fibrosis transmembrane conductance regulator (CFTR)-mediated chloride transport and ciliary beating of ciliated sinonasal epithelium cells, which could support this idea [38]. As for quercetin and some other flavonoids, an inhibition of PDEs was reported [31,34], it is likely that the observed effects are due to this mode of action. Since flavonoids are known to be poorly bioavailable after an oral intake [39], drug administration through inhalation of lyophilized and pulverized *Drosera* extracts should be taken into consideration. Of note, in in vivo experiments, a reduction in methacholine-induced airway resistance by nebulized quercetin was demonstrated [31], indicating that beneficial effects on ciliary beating might be possible by this way of administration. Standardized extracts containing defined amounts of quercetin and 2″-*O*-galloylhyperoside should be used for further pharmacological experiments since *Drosera* spp. differ in flavonoid composition with respect to quality and quantity [4].

In conclusion, our study shows that extracts, fractions and pure compounds from *Drosera rotundifolia* exert pharmacological effects on the musculature of the lower respiratory tract and ciliary beating. It is likely that extracts provide beneficial effects during a common cold by reducing spasms of ASM. Furthermore, the accelerated CBF identified as another mode of action for quercetin and 2″-*O*-galloylhyperoside is expected to improve mucociliary clearance, supporting an important innate defense mechanism of the lower airways.

## 4. Materials and Methods

### 4.1. Chemicals and Reagents

Solvents were of analytical quality and obtained from VWR International (Darmstadt, Germany). Acetylcholine chloride (purity >99%), quercetin (purity >95%) and 8MmIBMX (purity >98%) were purchased from Sigma-Aldrich (Steinheim, Germany). The 2″-*O*-galloylhyperoside used for contraction experiments and measurements of ciliary beating (purity >98%) was isolated from *D. rotundifolia* as described below. The 2″-*O*-galloylhyperoside (purity >98%) tested in the PDE assays and used for quantification was purchased from Biopurify Phytochemicals (Chengdu, China). Hyperoside (purity >95%) was purchased from Carl Roth (Karlsruhe, Germany). Rolipram (purity >98%) was obtained from Biotrend (Cologne, Germany).

### 4.2. Plant Material and Extraction

Drosera herbal plant material was collected in Rastede (Germany, 53°15′59.9″ N 8°15′01.3″ E) and identified as *Drosera rotundifolia* L. by Dr. J. Schulz. The extraction process was executed according to method 1.1.3 of the European Pharmacopoeia [2], which is used for mother tinctures of *D. rotundifolia*. The amount of ethanol 50% (*v/v*) was calculated to receive 1.2% of dry residue, as required in the monograph of the German Homeopathic Pharmacopoeia [3]. For better reproducibility, handling, stability and in deviation from the extraction process described in the European Pharmacopoeia, the liquid was removed using a rotary evaporator to obtain a dry extract. The lyophilisate was solved in acetonitrile/water (1:1, *v/v*) for phytochemical characterization of the dry extract. For pharmacological investigations, Krebs–Henseleit buffer (in mM: NaCl 118.1, KCl 4.7, CaCl_2_ 2.5, MgSO_4_ 1.2, KH_2_PO_4_ 1.2, NaHCO_3_ 25, glucose 5.6, pH of 7.4) supplemented with 0.3 or 1% DMSO depending on the final concentration of the extract was used. Preparations containing organic solvents are referred to as “DE″. For the preparation of an aqueous fraction (DF_A_), the dry extract was solved in DMSO-free Krebs–Henseleit buffer and centrifuged before use to remove insoluble compounds (1000 rpm, 10 min, 22 °C).

### 4.3. Phytochemical Characterization of the Drosera Dry Extract

UHPLC-ESI-qTOF-MS analysis was performed using a Dionex Ultimate 3000 RS Liquid Chromatography System with an AcclaimTM RSLC 120 C18 (2.1 × 100 mm, 2.2 μm) column (ThermoFisher Scientific, Waltham, MA, USA) at 40 °C. Injection volume: 2 µL. Flow rate: 0.5 mL/min. Samples were dissolved in acetonitrile/water 1:1 (*v/v*) at a concentration of 12.34 mg/mL. The binary gradient comprised of (A) water with 0.1% formic acid and (B) acetonitrile with 0.1% formic acid. Gradient: 0.00 to 1.12 min isocratic at 10% B; 1.12 to 4.12 min linear to 20% B; 4.12 to 10.12 min linear to 55% B; 10.12 to 11.62 min linear to 100% B; 11.62 to 15.00 min isocratic at 100% B; 15.00 to 15.10 min linear to 10% B; 15.10 to 20.00 min isocratic at 10% B. Eluted compounds were detected using a Dionex Ultimate DAD-3000 RS (*λ* = 200 to 800 nm) and a Bruker Daltonics micrOTOF-QII time-of-flight mass spectrometer with an Apollo electrospray ionization source in positive mode at 3 Hz over a mass range of *m/z* 50–1500. The UV chromatogram at λ = 280 nm and the base peak chromatogram (representing the most intense peaks at any time) [20] of the DE are displayed in Figure 1. The major peaks of the base peak chromatogram were identified by comparison of the *m/z* values from the [M + H]^+^ ion and fragments in the respective MS^2^ spectra with the respective datasets from the literature (Table 1).

### 4.4. Isolation and Identification of 2″-O-galloylhyperoside

An amount of 230 g of fresh plant material was pulverized with liquid nitrogen. Ethanol 90% (*v/v*) was added, and the plant was extracted with an Ultra-Turrax rotor stator system under ice cooling (9500 rpm, 15 min). Then, the liquid extract was filtered, concentrated and lyophilized. Fast centrifugal partition chromatography FCPC (Gilson, Middleton, WI, USA) was used to fractionate the ethanol 90% extract from *D. rotundifolia*. For each fractionation procedure, 0.5 g of the dry extract was dissolved in 5 mL ethyl acetate/water 1:1 (*v/v*). Mobile phase: Ethyl acetate (saturated with water), stationary phase: water (saturated with ethyl acetate), ascending mode: 195 × *g*; 10 mL/min, fraction size: 5 mL. Selected fractions were purified via column chromatography on Sephadex^®^ LH-20 (column size 130 × 29 mm i.d.) by isocratic elution with ethanol/water 1:1 (*v/v*) to isolate 2″-*O*-galloylhyperoside (yield: 51.4 mg).

Identity was determined by mass spectrometry, followed by ^1^H and ^13^C NMR. NMR spectra were recorded on an Agilent DD2 spectrometer (Agilent Technologies, Santa Clara, USA) at 600 MHz (^1^H) or 150 MHz (^13^C). The sample was dissolved in methanol-*d*_4,_ and chemical shifts were referenced to the respective residual solvent signals (3.31; 49.00 ppm).

Measured data were in accordance with those reported in the literature [4]: ESI-qTOF-MS *m/z* 617.1160 [M + H]^+^ (calculated for C_28_H_25_O_16_, 617.1137). ^1^H NMR (CD_3_OD, 600 MHz) δ 7.64 (1H, d, *J* = 2.3 Hz; H-2′), 7.50 (1H, dd, *J* = 8.5, 2.2 Hz; H-6′), 7.13 (2H, s; H-6′″/2′″), 6.78 (1H, d, *J* = 8.5 Hz; H-5′), 6.33 (1H, d, *J* = 2.2 Hz; H-8), 6.16 (1H, d, *J* = 2.1 Hz; H-6), 5.68 (1H, d, *J* = 7.9 Hz; H-1′’), 5.45 (1H, dd, *J* = 10.0, 7.9 Hz; H-2′’), 3.93 (1H, d, *J* = 3.4 Hz; H-4′’), 3.82 (1H, dd, *J* = 9.9, 3.4 Hz; H-3′’), 3.69 (2H, m; H-6′’), 3.59 (1H, t, *J* = 6.2 Hz; H-5′’). ^13^C NMR (CD_3_OD, 150 MHz) δ 179.1 (C-4), 168.2 (C-7′″), 166.1 (C-7), 163.1 (C-5), 158.3 (C-8a), 158.0 (C-2), 149.8 (C-4′), 146.3 (C-3′″/5′″), 145.9 (C-3′), 139.9 (C-4′″), 135.0 (C-3), 123.1, 123.0 (C-1′), 121.6 (C-1′″), 117.1 (C-2′), 116.2 (C-5′), 110.6 (C-6′″/2′″), 105.7 (C-4a), 101.2 (C-1′’), 99.8 (C-8), 94.6 (C-6), 77.5 (C-5′’), 74.5 (C-2′’), 73.5 (C-3′’), 70.6 (C-4′’), 62.0 (C-6′’).

### 4.5. UHPLC-PDA Analysis: Quantification of 2″-O-galloylhyperoside, Quercetin and Hyperoside

*Drosera* dry extract was dissolved in acetonitrile/H_2_O, 1:1, *v/v*, (DE) or Krebs–Henseleit buffer (DF_A_) and analyzed at a concentration of 0.5 mg/mL via Waters Acquity UPLC system on an Acquity UPLC HSS T3 (1.8 µm, 2.1 × 100 mm) stationary phase (Waters, Milford, U.S.A.). Mobile phase: (A) water with formic acid 0.1%; (B) acetonitrile with 0.1% formic acid; gradient: 0 to 2 min: isocratic at 10% B; 2 to 5 min: linear to 20% B; 5 to 11 min: linear to 45% B; 11 to 12.5 min: linear to 100% B; 12.5 to 14.5 min: isocratic at 100% B; 14.5 to 15 min: linear to 10% B. Column temperature: 40 °C. Flow rate: 0.5 mL/min. Injection volume: 2 µL. Compounds were detected using a Waters Acquity PDA eλ (λ = 200 to 800 nm) and a Waters Acquity QDa detector in positive ionization mode (cone voltage of 15 V, capillary voltage of 0.8 V). For quantification, the respective UV maxima were used (2″-*O*-galloylhyperoside: *λ* = 359 nm, hyperoside: *λ* = 355 nm, quercetin: *λ* = 370 nm). For determination of the amount of 2″-*O*-galloylhyperosid, a four-point calibration, and for quercetin and hyperoside, a five-point calibration curve with standards (double injection) was used. Samples of DE and DF_A_ were measured as independent triplicates (*n* = 3). Representative UV chromatograms of DE and DF_A_ at λ = 360 nm are displayed in Figure 2C and Figure 3D.

### 4.6. Animals and Dissection of Mouse Tracheal Slices

Male and female C57BL/6N mice (Charles River, Sulzfeld, Germany, and own breeding at the Institute of Pharmaceutical and Medicinal Chemistry, University of Münster, Germany) were housed under standard conditions. Animal care followed the rules of German laws (Az. 53.5.32.7.1/MS-12668, Health and Veterinary Office Münster, Germany). The mice were sacrificed using CO_2_.

### 4.7. Tissue Bath Experiments

A Mayflower horizontal tissue bath (Hugo Sachs Elektronik, Germany) was used to measure contractions. Murine tracheal slices of 3 mm were placed in the 5 mL tissue bath at a preload of 0.2 cN. Krebs–Henseleit buffer (37 °C) was continuously gassed (O_2_:CO_2_ 95:5). *Drosera* dry extract (0.5 mg/mL) was solved in Krebs–Henseleit buffer containing 1 % DMSO (DE). DF_A_ was obtained as described above. Hyperoside, 2″-*O*-galloylhyperoside and quercetin were dissolved in Krebs–Henseleit buffer; for quercetin, 0.5% DMSO was added to ensure solubility up to the highest concentration (100 µM). Control solutions contained equal amounts of DMSO depending on the respective test compound. Experiments were started after an equilibration time of 60 min. Tracheal tension was measured isometrically (force transducer F10 Type 375, amplifier TAM-A Type 705/1, evaluation software HSE ACAD 2.0, Harvard Apparatus GmbH, March-Hugstetten, Germany). The last ACh-induced contraction (100 µM) before adding test compounds was set as 100%. Test solutions were added 10 min before the next ACh bolus. When using a high potassium solution (60 mM K^+^), NaCl concentration was reduced to maintain osmolarity. At least 3 K^+^-induced contractions (each 20 min) were averaged and taken as control contraction. The maximal contraction represents the highest value, while the plateau (plat.) was calculated in minutes 19–20. IC_50_ values were calculated by the fitting of sigmoidal dose–response curves with a variable slope using GraphPad Prism version 3.00 for Windows (GraphPad, San Diego, CA, USA).

### 4.8. Measurements of the Ciliary Beat Frequency (CBF)

Murine tracheal explants were placed in LHC-9 medium (Invitrogen, CA, USA), cut into 0.5 mm slices and stored at 37 °C and an atmosphere of 5% CO_2_. The medium was changed every 3 days. CBF was measured up to 7 days after dissection. Slices were placed in a 0.7 mL perfusion chamber at 37 °C and perfused with Krebs–Henseleit buffer (1 mL/min). The tissue bath was located at an inverted Nikon Eclipse Ti2 microscope (Nikon GmbH, Düsseldorf, Germany) with a 100× oil objective. *Drosera* dry extract (0.2 mg/mL) was solved in Krebs–Henseleit buffer containing 0.3% DMSO (DE). The amount of DMSO was not exceeding 0.3%; as for this concentration, no effects on CBF were reported [40]. DF_A_ was prepared as described above. Quercetin, 8MmIBMX, and rolipram were solved in Krebs–Henseleit buffer containing 0.1% DMSO. 2″-*O*-Galloylhyperoside, ATP and salbutamol were solved without DMSO. In case DMSO was added, all test solutions of one experiment contained the same amount of DMSO. For calculation of the CBF, videos were recorded with a high-speed (200 frames per second) Basler aceA1300–200um camera (BaslerVision Technologies, Ahrensburg, Germany) after an equilibration period of 20 min. Videos were captured and analyzed by the SAVA software (Ammons Engineering, Clio, MI, USA) [41]. Ciliated cells exerted baseline frequencies in a range of 5 to 20 Hz, in agreement with the literature [42]. Data were normalized to values of the last 5 min before changing to a test solution (CBF_basal_), and effects on ciliary beating were expressed as a ratio (CBF_Ratio_).

### 4.9. Phosphodiesterase Inhibitor Assays

The activity of PDE4D and PDE1A was measured with assay kits #60345 and #60310 from Biomol (Hamburg, Germany). Assays were based on fluorescence polarization using fluorescein amidites-labeled cAMP and were performed according to the manufacturer’s instructions. Samples were recorded as duplicates in a 96-well plate containing equal amounts of DMSO (1%). The fluorescence polarization was measured at excitation wavelengths between 480 and 516 nm and emission wavelengths between 530 and 540 nm by using the plate reader CLARIOstar (BMG Labtech, Ortenberg, Germany). The IC_50_ values were calculated by a fitting of sigmoidal dose–response curves (variable slope) with GraphPad Prism version 3.00 for Windows (GraphPad, San Diego, CA, USA).

### 4.10. Statistics

Tissue bath experiments were performed using tracheal slices of 3 to 5 different mouse preparations. Measurements of CBF were carried out by using tracheal slices of at least 3 different preparations, while a single measurement represents an independent experiment. All results are expressed as the means ± SD. Different conditions were compared by a two-tailed paired Student’s *t*-test, while ANOVA (analysis of variance) followed by Student–Newman–Keul’s post hoc test was used for multiple comparisons. The null hypothesis of single experiments was that extracts or test solutions do not differ in the respective parameter. Statistical significance is considered if *p*-values are ≤0.05. Confidence intervals (CI) were used when IC_50_ values were calculated.

## Figures and Tables

**Table 1:** LC-qTOF-MS peak characteristics of *Drosera* dry extract (DE). Peaks were assigned to known constituents of the genus *Drosera* as denominated in the references. Assignments without references are based on the de novo interpretation of MS, MS^2^ and UV-spectra.

Peak No.	t_R_ (min)	*λ*_max_ (nm)	[M+H]^+^ (*m/z*)	MS^2^ Fragments (*m/z*)	Ion Formula	Error (mDa)	mSigma	Tentative Compound Identity	Ref.
1	2.377	-	221.0676	203.0558, 175.0605, 157.0409, 129.0178, 115.0398	C_8_H_13_O_7_	−2.0	54.2	1,5-Dimethylcitrate	
2	4.654	220, 272	199.0608	171.0270, 153.0166	C_9_H_11_O_5_	−0.6	1.6	Ethyl gallate	
3	5.557	216, 270, 357	633.1103	319.0437, 315.0719, 153.0167, 127.0427	C_28_H_25_O_17_	−1.6	4.3	Myricetin-3-*O*-(6″-*O*-galloyl)-hexoside	[21]
4	5.641	212, 269, 360	779.1669	617.1122, 477.1330, 315.0704, 303.0497, 153.0156	C_34_H_35_O_21_	−0.3	10.5	tentatively identified Quercetin- (galloylhexosyl)-hexoside	
5	6.186	214, 252, 363	303.0142	285.0069, 275.0145, 257.0081, 247.0264, 229.0135, 219.0262, 201.0202, 173.0215,	C_14_H_7_O_8_	0.6	18.8	Ellagic acid	[21]
6	6.435	212, 259, 356	465.1036	303.0496	C_21_H_21_O_12_	0.9	7.5	Hyperoside	[4]
7	6.736	216, 264, 356	617.1173	303.0527, 233.0455, 153.0181	C_28_H_25_O_16_	0.6	106.1	2″-*O*-galloylhyperoside	[4]
8	7.455	216, 268, 354	601.1183	315.0710, 287.0545, 233.0410, 205.0485, 153.0160	C_28_H_25_O_15_	0.5	16.6	Kaempferol-3-*O*-(2-*O*-galloyl)-*β*- galactopyranoside	[4,5]
9	7.6480	216, 262, 360	601.1191	465.1033, 303.0501, 299.0769, 137.0214	C_28_H_25_O_15_	0.3	10.4	tentatively identified Quercetin- (dihydroxybenzoyl-)hexoside	
10	7.759	212, 256, 368	319.0441	273.0387, 245.0431, 217.0476, 179.0311, 165.0150, 153.0150	C_15_H_11_O_8_	−0.8	1.8	Myricetin	
11	5.741	206, 262, 364	585.1246	303.0509, 283.0835, 265.0676, 121.0268	C_28_H_25_O_14_	−0.7	8.8	tentatively identified Quercetin- (hydroxybenzoyl-)hexoside	
12	8.846	212, 256, 368	611.1389	309.0973, 303.0471, 147.0428	C_30_H_27_O_14_	0.7	8.3	tentatively identified Quercetin- (cumaroyl-)hexoside	
13	8.956	220, 256, 368	303.0497	229.0487, 153.0161, 137.0223	C_15_H_11_O_7_	0.7	37.6	Quercetin	[4]
14	9.222	220, 243, 371	331.0463	316.0227, 300.9989, 271.0255, 253.0097	C_16_H_11_O_8_	0.8	3.8	3,3′-Di-*O*-methylellagic acid	[4]
15	9.699	220, 269, 356	569.1301	303.0504, 267.0856, 123.0420, 105.0330	C_28_H_25_O_13_	−1.2	6.3	Quercetin-(benzoyl-)hexoside	[21]
16	9.910	220, 265, 362	287.0558	213.0574, 165.0160, 153.0168, 121.0290	C_15_H_11_O_6_	−0.1	35.0	Kaempferol	[4]
17	10.075	220, 268, 355	553.1359	287.0558, 267.0863, 123.0409, 105.0338	C_28_H_25_O_12_	−1.8	11.4	tentatively identified Kaempferol-(benzoyl-)hexoside	
18	10.157	220, 268, 355	553.1374	287.0546, 267.0870, 123.0462, 105.0334	C_28_H_25_O_12_	−3.3	55.3	tentatively identified Kaempferol-(benzoyl-)hexoside	

**Table 2:** Quantification of the flavonoids 2″-*O*-galloylhyperoside (GH), quercetin (Q) and hyperoside (H) in DE and DF_A_ at a concentration of 0.5 mg/mL by UHPLC-PDA.

Sample	GH (µM)	Q (µM)	H (µM)
DE	88 ± 3	71 ± 30	1.8 ± 0.2
DF_A_	67 ± 5	9 ± 3	2.3 ± 0.3

**Table 3:** Inhibition of the PDE1A and PDE4D by the flavonoids 2″-*O*-galloylhyperoside (GH), quercetin (Q) and hyperoside (H) and the respective controls.

	GH IC_50_ (µM)	Q IC_50_ (µM)	H IC_50_ (µM)	8MmIBMX IC_50_ (µM)	Rolipram IC_50_ (µM)
PDE1A	3.0 (CI: 2.8–3.3)	4 (CI: 3–5)	not tested	12 (CI: 9–18)	−
PDE4D	1.1 (CI: 0.7–1.5)	2 (CI: 2–3)	3 (CI: 3–4)	−	0.6 (CI: 0.5–0.7)

## Data Availability

Raw data are available at the Institute of Pharmaceutical and Medicinal Chemistry (University of Münster, Germany).

## Sample Availability

A voucher specimen (DE 001) of the *Drosera* dry extract is deposited at the archives of the Institute of Pharmaceutical and Medicinal Chemistry (Münster, Germany).
